# Irisin participates in the beneficial effects of exercise in preventing gestational diabetes mellitus in overweight and obese pregnant women and a mouse model

**DOI:** 10.3389/fnut.2022.1034443

**Published:** 2023-01-19

**Authors:** Chen Wang, Xiaoming Zhang, Minghui Liu, Shengtang Qin, Chengrong He, Yingnan Liu, Jing Huai, Qidi Zhang, Yumei Wei, Huixia Yang

**Affiliations:** Department of Obstetrics and Gynecology, Peking University First Hospital, Beijing, China

**Keywords:** exercise intervention, GDM, irisin, overweight and obese, pregnant, mouse model

## Abstract

**Objective:**

We aimed to explore whether irisin participates in the beneficial effects of exercise in preventing gestational diabetes mellitus (GDM) in overweight and obese pregnant women.

**Study design:**

Sixty overweight and obese pregnant women each in the exercise and control groups were randomly selected from our previous randomized controlled trial. Eighteen obese model mice were generated and divided into exercise and control groups in which body weight, abdominal circumference, anal temperature, glucose tolerance test, and insulin tolerance test were recorded. The plasma irisin level, the expression of *PGC-1*α*/FNDC5* and brown (*UCP1*) and beige adipose (*CD137*, *TMEM26*, and *TBX-1*) marker genes were detected in muscle and adipose tissue.

**Results:**

In the human study, women in the exercise group had a significantly higher irisin level and lower insulin resistance level than those in the control group. Enhanced expression of beige adipose tissue marker genes (*CD137*, *TMEM26*, and *TBX-1*) in omental adipose tissue and the *CD137* gene in subcutaneous adipose tissue were found in the exercise group compared to the control group. In a mouse model, body weight and abdominal circumference were decreased, while glucose homeostasis and insulin sensitivity were significantly improved, and anal temperature was elevated after exercise intervention. A significantly higher level of irisin was revealed in the exercise group after undergoing exercise treatment. The expression of the beige adipose marker genes *CD137* and *TBX-1* was significantly higher in the exercise group than in the control group in posterior subcutaneous adipose tissue from the inguinal area and interscapular adipose tissue respectively.

**Conclusion:**

Our observations show that regular exercise during pregnancy can increase irisin levels, promote white fat beiging/browning, improve glucose homeostasis and enhance body energy expenditure, which may be one of the mechanisms by which exercise prevents GDM.

## Introduction

Gestational diabetes mellitus (GDM), defined as glucose intolerance with first onset in pregnancy, excluding diabetes mellitus (DM) before pregnancy, is considered the most common metabolic disturbance during pregnancy, leading to various adverse pregnancy outcomes for both mothers and offspring, not only during pregnancy but also in the long term (1–5). Research in the past has demonstrated that there are numerous well-documented risk factors for GDM (6). Among them, being overweight or obese is the most widely accepted risk factor (6–8), which can triple its occurrence (9, 10). Although the etiology of GDM is complex, insulin resistance (IR), caused by reduced insulin sensitivity in adipose tissue, muscle and liver, is a primary feature (6, 11).

Exercise, as an important part of lifestyle intervention, has been proven to be an important method for weight control and an effective way to prevent and manage metabolic disorders. Our previous randomized controlled trial (RCT) reported that intensive exercise intervention during pregnancy can significantly reduce the risk of GDM in overweight and obese pregnant women (exercise group vs. control group: 22.0% vs. 40.6%, OR 0.412, 95% CI, 0.240–0.705, *P* < 0.001) (12). However, the mechanism by which exercise prevents GDM is not well understood.

Many researchers assume that skeletal muscle is not only a locomotive organ but also an endocrine organ that can secrete myokines as a type of hormone (13, 14). Irisin is one of the myokines that is secreted into the circulation following the upregulation of proliferator-activated receptor-γ coactivator 1α (*PGC-1*α) in muscle through exercise and proteolytic cleavage from fibronectin-type III domain-containing 5 (*FNDC5*) (15). Previous studies have demonstrated that irisin plays an important role in white adipose tissue (WAT) beiging and browning (15), consequently inhibiting the accumulation of adipose tissue, increasing total body energy expenditure and improving obesity-linked insulin resistance (16–18).

Therefore, we aimed to explore whether irisin plays a role in the beneficial effects of exercise in preventing the occurrence of GDM in overweight and obese pregnant women.

## Materials and methods

### Subjects

#### Humans

Based on our previous RCT study (NCT02304718) (12), a total of 120 patients were chosen randomly from the clinical cohort, including 60 cases each in the exercise and control groups. The study protocol and exercise intervention of the RCT were reported previously (12). Briefly, 300 overweight and obese (BMI ≥28 kg/m^2^) pregnant women at <12^+6^ gestational weeks were recruited and randomly allocated to either an exercise or a control group. Pregnant women allocated to the exercise group performed cycling exercise with moderate intensity 3 times per week (at least 30 min/session with a rating of perceived exertion between 12 and 14) that was initiated within 3 days of randomization until 37 gestational weeks. Those in the control group continued their usual daily activities.

#### Animals

Female 5-week-old C57BL/6J mice of specific pathogen-free (SPF) grade were purchased from WeiTongLiHua Experimental Animal Technology Co., Ltd., Beijing, China. All mice were housed four per cage in an Office of Laboratory Animal Welfare-certified animal facility with a 12-h light, 12-h dark cycle. Animal protocols were reviewed and approved by the Institutional Animal Care and Use Committee of Peking University First Hospital (J201935). Using independent ventilation cages, all mice were divided into a high-fat diet (HFD)-fed group (45% fat, 20% protein, and 35% carbohydrates) and a normal diet-fed group (12% fat, 24% protein, and 64% carbohydrates; Beijing KeAoXieLi Feeds Co., Ltd., Beijing, China). After 12–18 weeks of feeding, 24 obese mice were successfully established. A mouse was considered obese when the degree of obesity [(HFD-fed group weight–normal diet-fed group weight)/normal diet-fed group weight × 100%)] was greater than 20%. Then, obese female mice were mated with male C57BL/6J mice of the same age. When the presence of a vaginal plug appeared after mating (defined as day 0 of pregnancy), pregnancy was considered successful. Finally, 18 mice were successfully mated and randomly divided into an exercise intervention group (*n* = 9) and a control group (*n* = 9). The pregnant mice in the exercise intervention group were required to undergo mandatory running wheel training every day, with a wheel speed of 10.0 rpm, running difficulty at medium, and duration of 60 min. The control group was not given any special intervention.

## Data collection and tissues

### Humans

Clinical and demographic characteristics of the 120 patients were extracted from our previous RCT study. During the RCT study, fasting venous blood (fasting time >8 h and <4 h) was collected at the beginning of the study (9–11 weeks of gestation), in the second trimester of pregnancy (25 weeks of gestation), and in the third trimester of pregnancy (36 weeks of gestation). Within 4 h after collection, the venous blood was centrifuged at 4°C for 10 min at 1,800 × g, plasma was separated and repacked, and it was stored at −80°C. For women with cesarean section (23 in the exercise group and 24 in the control group), rectus abdominis, omental adipose tissue, and subcutaneous adipose tissue were collected immediately after delivery in the operating room. All samples were washed twice with sterile normal saline, placed into an RNase-inactivated cryopreservation tube, immediately placed in liquid nitrogen for quick freezing, and then stored in a freezer at −80°C.

### Mice

Blood was obtained on day 0 of pregnancy. Weight, abdominal circumference, food intake, and anal temperature were collected every 3 days. A glucose tolerance test (GTT) was performed on day 13 of pregnancy, and an insulin tolerance test (ITT) was performed on day 18 of pregnancy. After sacrifice on day 19.5 of pregnancy, blood was obtained from the abdominal aorta, centrifuged at 3,000 × *g* for 10 min and stored at −80°C until analysis. Rectus femoris, posterior subcutaneous adipose tissue from the inguinal area and interscapular adipose tissue were carefully dissected, weighed, immediately frozen in liquid nitrogen and stored at −80°C.

**FIGURE 1:**
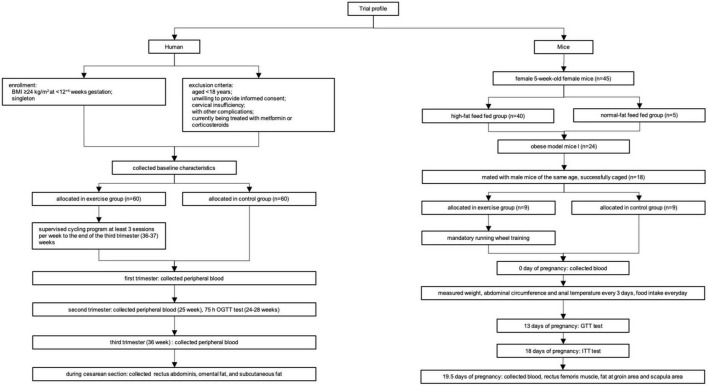
The flowchart of the experiments.

The flowchart of the experiments is shown in Figure 1.

### Methods

#### Irisin level

Human plasma irisin and mouse plasma irisin were detected with a commercially available ELISA kit (Phoenix Co., Ltd., CANO: EK-067-29, USA). The detection range of irisin is 0.1–1000 ng/mL.

#### Gene expression analysis

Expression of the *FNDC5* and *PGC-1*α genes was detected in rectus abdominis muscle in humans and in rectus femoris muscle in mice. Expression levels of the brown adipose marker gene *UCP1* and beige adipose marker genes *CD137*, *TMEM26*, and *TBX-1*, and *PGC-1*α were detected in omental and subcutaneous adipose tissue in humans and posterior subcutaneous adipose tissue from the inguinal area and interscapular adipose tissue in mice (15, 17, 19). According to the manufacturer’s protocol, total RNA was isolated from 100 mg of tissue using 1 mL of TRIzol Reagent (Invitrogen, USA). Two micrograms of total RNA was used as a template for reverse transcription. Oligo (dT) 10 primers and reverse transcriptase (Applied Biosystems, USA) were used. cDNA quantity was measured using real-time PCR with the ABI PRISM 7500 sequence detector system. PCR was performed in a final volume of 20 μl, consisting of diluted cDNA sample (2 μg), 5× All-In-One RT MasterMix Kit (Abmgood Co., Ltd., Canada), primers and primer probes optimized for each target gene and nuclease-free water. All samples were analyzed in triplicate. Primers were designed using Primer Express 3.0 software. For humans, the following primers were used: *FNDC5*, sense 5′-TGCAGGCCATCTCCATTCAG-3′, antisense 5′- TGAACAGGACCACGACGATG-3′; *PGC-1*α, sense 5′-ACAG CTTTCTGGGTGGATT-3′, antisense 5′-TGAGGACCGCTAG CAAGTTT-3′; UCP1, sense 5′-ACCGCAGGGAAAGAAACA GC-3′, antisense 5′-TCAGATTGGGAGTAGTCCCT-3′; *CD1 37*, sense 5′-AGCTGTTACAACATAGTAGCCAC-3′, antisense 5′-TCCTGCAATGATCTTGTCCTCT-3′; *TMEM26*, sense 5′-A TGGAGGGACTGGTCTTCCTT-3′, antisense 5′-CTTCACCT CGGTCACTCGC-3′; and TBX1 sense 5′-ACGACAACGGCCA CATTATTC-3′, antisense 5′-CCTCGGCATATTTCTCGCTAT CT-3′. For mice, the following primers were used: FNDC5, sense 5′-GAAGAAGGATGTGCGGATGCTC-3′, antisense 5′-C TGTCCCTGGATGGAGATGG-3′; *PGC-1*α, sense 5′-AGCCGT GACCACTGACAACGAG-3′, antisense 5′-GCTGCATGGTTC TGAGTGCTAAG-3′; *UCP1*, sense 5′-ACTGCCACACCTCCA GTCATT-3′, antisense 5′-CTTTGCCTCACTCAGGATTGG-3′; *CD137*, sense 5′-CGTGCAGAACTCCTGTGATAAC-3′, antisense 5′-GTCCACCTATGCTGGAGAAGG-3′; *TMEM26*, sense 5′-ACCCTGTCATCCCACAGAG-3′, antisense 5′-TGTT TGGTGGAGTCCTAAGGTC-3′; and *TBX1* sense 5′-GGCAGG CAGACGAATGTTC-3′, antisense 5′-TTGTCATCTACGGGC ACAAAG-3′.

### Infrared thermography in mice

In the exercise group, infrared thermal images were taken on day 0 and day 19.5 using an infrared digital thermographic camera (Fortric 225S, Infrared Thermal Imaging Camera; FOTRIC, Japan) and analyzed by AnalyzIR software.

#### Glucose tolerance test and insulin tolerance test in mice

For the GTT, mice were fasted for 16 h (17:00 to 9:00), and their water intake was unrestricted. Glucose solution (2 g/kg body weight) was injected into the intraperitoneal cavity. Blood glucose levels were collected before, at 1 h, and at 2 h after injection. For the ITT, mice were fasted for 4 h (9:00 to 13:00), and their water intake was unrestricted. Insulin solution (0.5 U/kg body weight) was injected into the intraperitoneal cavity. Blood glucose levels were collected before and at 0.5, 1 and 2 h after injection.

### Statistical analysis

Continuous variables were tested for normality using the Shapiro-Wilk test, and non-parametric tests were used when appropriate. Two-tailed unpaired Student’s *t*-test, one-way ANOVA and the non-parametric Mann–Whitney *T* test were performed accordingly. For repeated measures, we also used a generalized estimating equation (GEE) to determine the significance of differences as estimated by main effects for groups (exercise vs. control) and group × time interaction effects. Statistical significance was defined as *p* < 0.05 or 0.01.

## Results

### Humans

The baseline characteristics of 120 subjects chosen from the RCT cohort were well matched between the exercise and control groups (Table 1). The incidence of GDM in the exercise and control groups was 20.0 and 36.7%, respectively, which represented a statistically significant decrease in the exercise group (*P* = 0.04). The characteristics of women with cesarean section in each group are also shown in Table 1. The age of women with cesarean section in the control group was significantly higher than that in the exercise group, while other baseline information was similar. The incidence of GDM in women with cesarean section in the exercise group was also significantly lower than that in the control group (21.7 and 50%, *P* = 0.04).

**TABLE 1:** Baseline characteristics of participants.

Characteristics	Exercise group total (*n* = 60)	Control group total (*n* = 60)	Exercise group Cs (*n* = 23)	Control group Cs (*n* = 24)	*p^a^*	*P^b^*
Age, years	31.58 ± 3.16	32.07 ± 3.95	31.52 ± 2.54	34.08 ± 4.14	0.46	0.01
Weight, kg	70.84 ± 7.31	71.35 ± 7.84	72.17 ± 8.01	70.97 ± 8.59	0.72	0.62
Height, cm	162.43 ± 4.73	162.70 ± 4.92	162.00 ± 5.04	161.83 ± 5.04	0.76	0.91
pBMI, kg/m^2^	26.86 ± 2.72	26.91 ± 2.26	27.49 ± 2.78	27.04 ± 2.27	0.91	0.54
Obese, BMI ≥28 kg/m^2^ (%)	18 (30.0)	16 (26.7)	10 (43.5)	7 (29.2)	0.69	0.31
Overweight 24≤ BMI ≤28 kg/m^2^	42 (70.0)	44 (73.3)	13 (56.5)	17 (70.8)	0.69	0.31
Maternal birthweight, g	3259.83 ± 475.19	3315.14 ± 557.05	3436.96 ± 487.62	3312.64 ± 691.68	0.56	0.49
≥College/university (%)	50 (83.3)	44 (73.3)	18 (78.3)	17 (70.8)	0.18	0.56
Gestational age, wk	10 ± 2	10 ± 2	10 ± 2	10 ± 2	0.27	0.31
Primiparous (%)	51 (83.3)	48 (80.0)	19 (82.6)	19 (79.2)	0.47	0.76
Family history of diabetes (%)	23 (38.3)	18 (30.0)	10 (43.5)	6 (25.0)	0.34	0.18
Fasting plasma glucose at study entry, mmol/L	5.05 ± 0.36	5.05 ± 0.37	5.06 ± 0.37	5.01 ± 0.44	0.98	0.65
Incidence of GDM (%)	12 (20.0)	22 (36.7)	5 (21.7)	12 (50.0)	0.04	0.04
GDM treatment					0.68	0.54
Lifestyle management	9 (75.0)	18 (81.8)	3 (60.0)	10 (83.3)		
Insulin	3 (25.0)	4 (18.2)	2 (40.0)	2 (16.7)		

pBMI, prepregnancy body mass index; GDM, gestational diabetes mellitus; Cs, cesarean section.

Continuous variables are presented as the mean ± SD; categorical variables are presented as *n* (%).

*p*^a^ represents exercise group (total, *n* = 60) vs. control group (total, *n* = 60); *P*^b^ represents exercise group (women with Cs, *n* = 23) vs. control group (women with Cs, *n* = 24).

**FIGURE 2:**
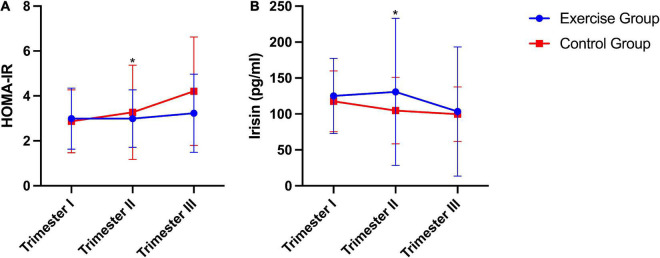
HOMA-IR and plasma irisin levels during pregnancy in humans. **(A)** HOMA-IR, homeostasis model assessment of insulin resistance index [fasting plasma insulin (mU/mL) × fasting glucose (mmol/L)/22.5]. **(B)** Plasma irisin level. The results are presented as the mean ± SD. *P < 0.05.

Significant differences in HOMA-IR [homeostasis model assessment of insulin resistance index, fasting plasma insulin (mU/mL) × fasting glucose (mmol/L)/22.5] (20) and plasma irisin levels between the two groups were found. The exercise group had a significantly lower HOMA-IR than the control group (*P* = 0.011). Conversely, plasma irisin levels were significantly higher in the exercise group than in the control group (*P* = 0.026). No significant group × time interaction effects were obtained from the HOMA-IR (*P* = 0.121) or plasma irisin level (*P* = 0.390) during pregnancy (Figure 2).

**FIGURE 3:**
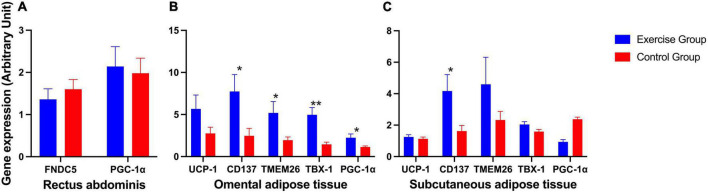
Expression of irisin-related genes and brown and beige adipose marker genes in humans. **(A)** Expression of irisin-related genes (*FNDC5* and *PGC-1*α) in human rectus abdominis. **(B)** Expression of the brown adipose marker gene *UCP-1* and beige adipose marker genes *CD137*, *TMEM26*, *TBX-1*, and *PGC-1*α in human omental adipose tissue. **(C)** Expression of the brown adipose marker gene UCP-1 and beige adipose marker genes *CD137*, *TMEM26*, *TBX-1*, and *PGC-1*α in human subcutaneous adipose tissue. The results are presented as the mean ± SE. *P < 0.05, **P < 0.01.

The expression of *FNDC5* and *PGC-1*α in the rectus abdominis showed no significant difference (Figure 3A). The expression of beige adipose marker genes (*CD137*, *TMEM26*, and *TBX-1*) was significantly enhanced in the exercise group compared to the control group in omental adipose tissue, so was the expression of *PGC-1*α. The expression of the *CD137* gene was also significantly enhanced in the exercise group compared to the control group in subcutaneous fat. However, the brown adipose marker gene *UCP1* showed no statistically significant difference in either omental or subcutaneous adipose tissue (Figures 3B, C).

### Animals

**FIGURE 4:**
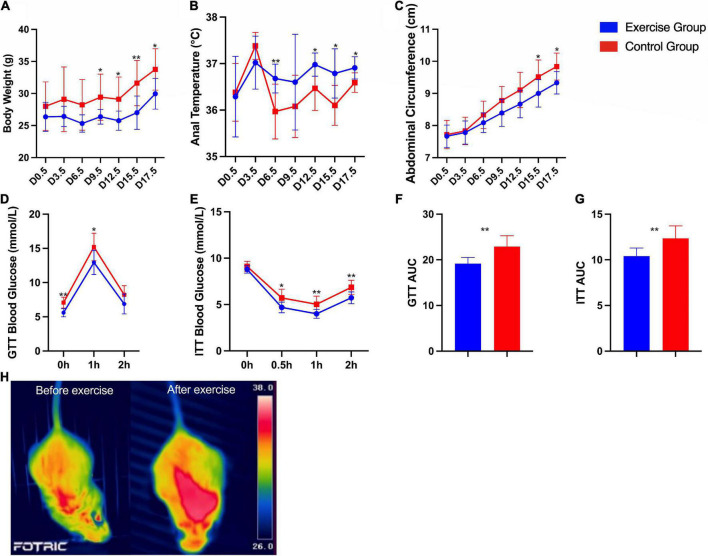
Characteristics and metabolic assessment of mice. **(A)** Body weight during pregnancy. **(B)** Anal temperature during pregnancy. **(C)** Abdominal circumference during pregnancy. **(D)** Glucose tolerance tests. **(E)** Insulin tolerance tests. **(F)** Area under the glucose tolerance test (GTT) curve. **(G)** Area under the insulin tolerance test (ITT) curve. **(H)** Infrared thermal images of a mouse in the exercise group before and after exercise. The results are presented as the mean ± SD. *P < 0.05, **P < 0.01.

Significant differences in body weight, anal temperature and abdominal circumference between the two groups were found. The body weight (*P* < 0.001) and abdominal circumference (*P* < 0.001) of the exercise group were significantly lower than those of the control group. The anal temperature (*P* < 0.001) of the exercise group was significantly higher than that of the control group. Significant group × time interaction effects were obtained for anal temperature (*P* = 0.005) and abdominal circumference (*P* < 0.001) during pregnancy. This result suggests that the anal temperature and abdominal circumference of mice in both groups increased over time, with the measures of anal temperature increasing more in the mouse in the exercise group and abdominal circumference increasing more in the mouse in the control group (Figures 4A–C).

Furthermore, glucose homeostasis and insulin sensitivity were significantly improved in the exercise group compared with the control group (Figures 4D–G). Infrared thermal images of a mouse in the exercise group on day 0 and day 19.5 are shown in Figure 4H as an example; these images show the significantly higher body temperature after exercise.

**FIGURE 5:**
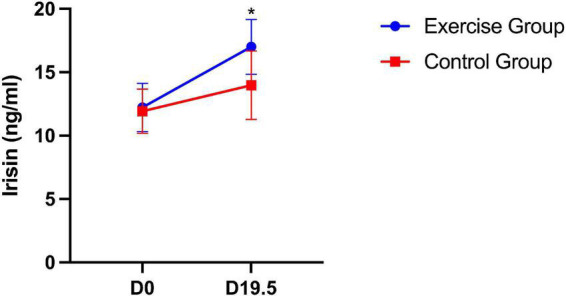
Plasma irisin level in mice on day 0 and day 19.5. The results are presented as the mean ± SD. *P < 0.05.

For plasma irisin levels in mice, significant differences between the two groups were found. The plasma irisin level was significantly higher in the exercise group than in the control group (*P* = 0.035). Significant group × time interaction effects were also obtained from the plasma irisin level during pregnancy (*P* = 0.012). This result suggests that the plasma irisin level of mice in both groups increased over time, with the level increasing more in the exercise group (Figure 5).

**FIGURE 6:**
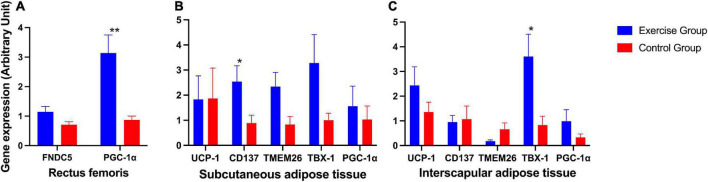
Expression of irsin-related genes and brown and beige adipose marker genes in mice. **(A)** Expression of irisin-related genes (*FNDC5* and *PGC-1*α) in the mouse rectus femoris. **(B)** Expression of the brown adipose marker gene *UCP-1* and beige adipose marker genes *CD137*, *TMEM26*, *TBX-1* and *PGC-1*α in mouse posterior subcutaneous adipose tissue from the inguinal area. **(C)** Expression of the brown adipose marker gene UCP-1 and beige adipose marker genes *CD137*, *TMEM26*, *TBX-1*, and *PGC-1*α in mouse interscapular adipose tissue. The results are presented as the mean ± SE. *P < 0.05, **P < 0.01.

The expression of the *PGC-1*α gene in the rectus femoris was significantly higher in the exercise group than in the control group (Figure 6A). In posterior subcutaneous adipose tissue from the inguinal area, the expression of the beige adipose marker gene *CD137* was significantly higher in the exercise group than in the control group (*P* = 0.023) (Figure 6B). In interscapular adipose tissue, the expression of the beige adipose marker gene *TBX-1* was significantly higher in the exercise group than in the control group (*P* = 0.015) (Figure 6C).

Other genes also showed an elevated trend in the exercise group compared to the control group, although these were not significantly different (Figures 6A–C).

## Discussion

Our study demonstrated a positive relationship between exercise and plasma irisin levels, exercise and white fat beiging/browning, and exercise and improvement in glucose homeostasis in both overweight and obese pregnant women and an obesity animal model. Thus, we hypothesize that the mechanism by which exercise prevents GDM may be by stimulating irisin secretion from skeletal muscle, thereby inducing beiging/browning and thermogenesis of WAT. This alteration leads to an increase in energy expenditure and an improvement in glucose homeostasis.

Gestational diabetes mellitus is defined as glucose intolerance that occurs during the second or third trimester of pregnancy, excluding DM before pregnancy (3). Throughout pregnancy, basal glucose production increases to supply fetal demand, while normal physiological IR is also elevated but subsequently disappears after delivery. More insulin is secreted by pancreatic beta cells to compensate for the change in glucose regulation and to therefore maintain blood glucose in a normal range (21). However, due to genetic or environmental factors, such as obesity, insulin receptor defects, placental hormones, and cytokine level abnormalities, IR is aggravated and becomes pathological IR (22). When the secretion of insulin cannot compensate for pathological IR, GDM occurs. Hence, the key to preventing GDM is to reduce pathological IR.

Overweight and obesity are some of the most salient risk factors for GDM. As reported by Kim et al. (23) the risk of GDM was almost two, three, and five times higher in overweight, obese and severely obese women, respectively. Enhanced IR with increasing BMI may explain this phenomenon, and this fact may be partly due to the change in balance between the relatively protective brown adipose tissue (BAT) and beige adipose and the deleterious WAT (24). In contrast to WAT, BAT, and beige adipose tissue, as thermogenic organs, have the ability to transfer energy from nutrients to heat, promote the uptake of glucose and improve insulin resistance. Stanford et al. (25) showed that transplantation of BAT into mice can improve their glucose tolerance, cause weight loss and thus reverse HFD-induced insulin sensitivity. Similarly, Shankar et al. (26) found that BAT transplantation in a mouse model of HFD-induced obesity reversed the HFD-induced increase in insulin resistance and attenuated obesity-associated adipose tissue inflammation. In contrast, extirpation of the interscapular BAT exacerbates HFD-induced obesity, insulin resistance and adipose tissue inflammation. In humans, cold-induced BAT activation also increases glucose uptake by ∼12-fold (27).

Irisin is a myokine that was first discovered by Boström et al. (15) during the process of exploring the mechanism of white fat beiging and browning caused by exercise. It has been reported that irisin can enhance the transformation from WAT to BAT and beige fat by upregulating beige and brown adipose marker genes (19). Moreover, when an anti-FNDC5 antibody is used to inhibit the activity of irisin, the transformation from white adipose to beige adipose tissue is also stopped (17).

Irisin levels have been proven to be lower in type 2 DM patients than in controls (28, 29), and irisin is thought to have an inverse relationship with insulin resistance. Thus, irisin is assumed to be an insulin enhancer (30). Furthermore, although the research is limited, most of the studies and a meta-analysis support that irisin levels are lower in pregnancies complicated with GDM. Notably, one prospective study also demonstrated that lower irisin levels in early pregnancy are an early marker for predicting GDM (31). Therefore, based on these findings, increasing irisin levels and upregulating browning and beiging of WAT can be a new therapeutic strategy to treat obesity and improve insulin sensitivity to prevent GDM in overweight and obese pregnant women.

As the most novel myokine, irisin can be induced by exercise. It was initially reported that plasma irisin levels were elevated by 65% after 3 weeks of free wheel running in mice and that there was a two-fold increase after 10 weeks of exercise training in healthy adult humans (15). Since then, irisin has become a research focus in exercise therapy for metabolic diseases, and subsequent studies have also confirmed that exercise is able to increase circulating irisin levels in individuals with and without metabolic syndromes (32, 33). However, the expression and secretion of irisin may be affected by different exercise intensities, types, times, and frequencies.

In our research, irisin levels were significantly increased in the exercise-enhanced group compared to the control group in the obesity mouse model. However, in humans, the difference in plasma irisin levels in the third trimester was weakened. The reason might be that (1) women in the control group who were diagnosed with GDM in the second trimester also underwent lifestyle intervention, including routine exercise and diet management in our hospital, which might attenuate the difference caused by exercise intervention between the two groups; (2) irisin slightly decreased with the development of pregnancy, as shown in a previous study (34) and in the control group in our study; thus, the exercise intervention performed in our human study may not have been able to raise irisin levels enough to compensate for the physiological decrease in irisin levels during pregnancy; and (3) irisin is not only a myokine but also an adipokine. Women in the control group had higher gestational weight gain than those in the exercise group, and thus, the non-significant difference in irisin levels between the two groups might partially be explained by the higher fat mass and presumably higher adipose tissue-derived irisin level in the control group in the third trimester (35).

Our study also showed that brown adipose and beige adipose marker genes were elevated in WAT in both overweight and obese women and in the obesity mouse model. Furthermore, in the mouse model, our study showed that body weight and abdominal circumference were decreased, while anal temperature was elevated during pregnancy in the exercise group compared to the control group. Furthermore, infrared thermal images of mice also showed significantly higher body temperature after exercise. In addition, glucose homeostasis and insulin sensitivity were significantly improved in the exercise group compared with the control group. Thus, based on both human and mouse model studies, the complete picture of the mechanism through which exercise prevents GDM may be by way of stimulating irisin secretion from skeletal muscle, thereby inducing beiging and browning and thermogenesis of WAT. This browning leads to an increase in energy expenditure and an improvement in glucose homeostasis.

Previous studies focusing on the potential mechanisms of exercise in preventing GDM have focused mainly on enhancing the expression and function of the insulin signaling pathway, regulating adipocytokines, modifying the gut microbiome, and reducing the inflammatory state and oxidative stress (36, 37). In the current study, we discuss the possible mechanism of exercise in preventing GDM during pregnancy from the perspective of irisin and WAT beiging and browning in overweight and obese populations based on both a clinical cohort and animal model. To the best of our knowledge, this is the first study to explore the potential mechanism of exercise in preventing GDM from the aspect of myokines and WAT beiging and browning. Importantly, our human study was based on an RCT study in which women with overweight and obesity in the exercise intervention group had high compliance, and the supervised cycling program ensured a moderate intensity of the exercise intervention. Furthermore, samples were collected prospectively. However, our human study centered on only Chinese overweight and obese women, so the conclusions may not be applicable to other ethnicities or populations. In addition, in our study, due to restrictions in human specimen collection, skeletal muscle could not be obtained. Thus, the negative results for *FNDC5* and *PGC-1*α gene expression in the human rectus shown in our study may not have scientific significance. However, skeletal muscle was obtained from the mouse model, and the results showed that the expression of both *FNDC5* and *PGC-1*α was increased in the exercise group compared to the control group, which partly compensated for the limitations in human research. Nevertheless, the sample size of the mouse model study was limited, which may be the reason why most BAT marker genes detected showed only an upward trend in the exercise group compared to the control group, while no statistically significant difference was found.

## Conclusion

In conclusion, we found that regular exercise during pregnancy can increase irisin levels, promote white fat beiging/browning and enhance body energy expenditure, which may be one of the mechanisms by which exercise improves glucose homeostasis and prevents GDM. However, the current lines of evidence of our study are mainly phenomenal descriptions, and the specific molecular mechanism of the interaction between exercise, irisin levels, white fat beiging/browning, and insulin resistance is still not clearly elucidated. Additionally, as the nature and secreted form of irisin are controversial, discrepancies must exist due to the differences in study populations, methods, detection techniques, and sample sizes. More high-quality and innovative studies are needed. Much information is still required to bridge our knowledge gap between exercise and the beneficial effects on metabolic diseases and to help provide new targets for treatment.

## Data availability statement

The raw data supporting the conclusions of this article will be made available by the authors, without undue reservation.

## Ethics statement

The studies involving human participants were reviewed and approved by the Ethics Committee of the Peking University First Hospital (2014[726]). The patients/participants provided their written informed consent to participate in this study. The animal study was reviewed and approved by the Ethics Committee of the Peking University First Hospital (J201935).

## Author contributions

CW developed the project, performed the experiments, analyzed data and wrote the manuscript. XZ, ML, SQ, CH, YL, and JH performed the experiments. XZ, YW, and QZ analyzed the data. HY developed the project and revised the manuscript. All authors contributed to the article and approved the submitted version.
